# Two regions, two lineages: Untangling two centuries of confusion between *Machilus glaucescens* and *M. macranthus* (Lauraceae) through integrative taxonomy

**DOI:** 10.1371/journal.pone.0357315

**Published:** 2026-09-09

**Authors:** Nabasmita Malakar, Ravikanth Gudasalamani, Ganesan Rengaian

**Affiliations:** 1 Manipal Academy of Higher Education, Manipal, Karnataka, India; 2 Ashoka Trust for Research in Ecology and the Environment (ATREE), Bangalore, India; 3 Sugadaira Research Station, Mountain Science Centre, University of Tsukuba, Nagano, Japan; USDA Forest Service Southern Research Station, UNITED STATES OF AMERICA

## Abstract

The Asian genus *Machilus* (Lauraceae) of the *Persea* group is widely distributed across South and Southeast Asia. Like many other genera of Lauraceae, *Machilus* has received limited systematic attention, particularly in India. As a result, the nomenclature and systematic positions of *Machilus glaucescens* (Nees) Wight and *M. macranthus* Nees have remained unclear. In this study, we employed an integrative taxonomic approach to disentangle the taxonomy, phylogenetic relationships and distributions of *M. glaucescens* (restricted to Indo-Burma biodiversity hotspot) and *M. macranthus* (restricted to Eastern Ghats and Western Ghats-Sri Lanka biodiversity hotspot). We analysed morphological, molecular, and distribution data to elucidate the systematic relationships following the mix-up of taxonomic status. We examined 28 morphological characteristics using cluster analysis and reconstructed phylogenies with two nuclear markers (*ITS* and *LEAFY* intron II). Additionally, we studied the distribution range of both species with ecological niche modelling. The combined evidence from nomenclature, morphological clustering, phylogenetic analyses, as well as the species distributional range limits confirms that *M. glaucescens* and *M. macranthus* are two distinct species with their current distributions restricted to separate biogeographic regions of Asia.

## Introduction

Accurate species delimitation through systematic research is fundamental for conserving biodiversity and genetic diversity. Determining whether taxa represent distinct species, varieties, or the same species matters because true species are typically reproductively isolated, especially when geographically separated, making correct classification essential to effective conservation planning and management. This study resolves a striking case of such taxonomic uncertainty involving two taxa within the genus *Machilus*.

*Machilus* is a species-rich genus within the *Persea* group [1], which currently comprises seven genera (*Alseodaphne*, *Alseodaphnopsis*, *Dehaasia*, *Machilus*, *Nothaphoebe*, *Persea* and *Phoebe*) of the plant family Lauraceae. The genus *Machilus* includes around 130 species, distributed in the evergreen forests of South and Southeast Asia.

Although *Machilus* species are widespread throughout Southeast Asia, their highest species diversity occurs in the region extending from the Himalayas to southern China. Economically, many species of *Machilus* are widely utilised for timber, essential oils, and in pharmaceutical products [2–4]. The fruit of *Machilus edulis* King ex Hook. f. is consumed as a wild edible fruit in the Sikkim state of Eastern Himalaya [5]. Additionally, the leaves of *Machilus gamblei* King ex. Hook.f. serve as a primary food source for muga silk moths, which produce the heritage muga silk cloth [6].

Over the past decades, significant advances in plant taxonomy have been achieved through the application of integrative taxonomic approaches [7–11]. Particularly, the incorporation of molecular data has greatly improved our understanding of phylogenetic relationships among taxa, as it provides insights on evolutionary relationships that are not evident from studies based on morphology alone [12,13]. By integrating morphological, molecular, or ecological evidence, integrative taxonomy improves classification accuracy and resolves species relationships that cannot be adequately addressed using a single aspect.

Two species of *Machilus* (*M. glaucescens* and *M. macranthus*) were recently considered as varieties under *M. macranthus* [14] as *M. macranthus* var. *glaucescens* and *M. macranthus* var. *macranthus*. However, these taxa occupy entirely distinct geographical ranges: *M. macranthus* var. *glaucescens* is distributed to the Indo-Burma biodiversity hotspot, whereas *M. macranthus* var. *macranthus* is distributed to the Western Ghats-Sri Lanka hotspot and Eastern Ghats hotspot of South India. Although we observed morphological differences between both taxa [see Table 1], the previous study [14] overlooked such differences as well as their geographical distributions, phylogenetic relationships, and nomenclature in declaring them as infraspecifics. Consequently, a comprehensive investigation incorporating an integrative taxonomic approach to determine their taxonomic status is warranted.

**Table 1:** Table of morphological differences between *M. glaucescens* and *M. macranthus.*

Characters	*M. glaucescens* (Nees) Wight	*M. macranthus* Nees
Distribution	Indo-Burma	Eastern Ghats, Western Ghats- Sri Lanka
Petiole	Pubescent	Glabrous
Leaf	Hairs fully covered the abaxial side when young, eventually confined only on veins in matured leaves, yellowish-brown glaucous when dry, leaf base equal	Glabrous on both sides, blackish glaucous when dry, leaf base unequal
Venation	Prominent on both sides, foveolate	Prominent on abaxial side, obscure along the adaxial side, non-foveolate
Paniculate inflorescence	Dense, with reddish brown hairs	Loosely packed, glabrous
Stamens	Filaments glabrous	Filaments hairy
Tepals in fruits	Spreading	Reflexed

Accurately resolving the taxonomic status of *M. macranthus* var. *glaucescens* and *M. macranthus* var. *macranthus* has significant conservation implications, recognizing as distinct species is crucial for accurate representations of the region’s biodiversity richness and phylogenetic diversity [15–17]. Integrating, specimen-based taxonomy, morphometric analysis, phylogenetics, and ecological modelling, we demonstrate that these two varieties represent distinct species belonging to two distinct lineages with significant divergence and complete geographical distinctness. Furthermore, because *M. macranthus* is the sole representative of the genus in the Western Ghats-Sri Lanka hotspot and Eastern Ghats, it demands attention on its own due to its evolutionary uniqueness and distinctiveness [15,18].

*M. glaucescens* and *M. macranthus* are frequently mixed up, with a long history of taxonomic confusion. This ambiguity may persist due to the lack of a combined assessment of names, protologues, geographical distribution and collection-based taxonomic approaches [14,19–23]. The nomenclature of *M. glaucescens* (Nees) Wight dates to 1814 when the name *Laurus glaucescens* Roxb. first appeared in *Hortus Bengalensis*, with its type location as Sylhet and vernacular name recorded as Orook [24]. Nees (1831), in *Plantae Asiaticae Rariores*, cited *Laurus glaucescens* Roxb. as α-variety within the genus *Ocotea* as species *O. glaucescens* [25]. Later, in *Systema Laurinarum* (1836), Nees treated *Ocotea glaucescens* Nees as synonym of *Phoebe glaucescens* (Nees) Nees [26].

Robert Wight (1852) transferred the species *Phoebe glaucescens* to the genus *Machilus*, publishing it as *Machilus glaucescens* (Nees) Wight, in *Icones Plantarum Indiae Orientalis* (*Ic. t.* 1825), citing the location as “Nilgherries, Western Slopes” [19]. He did not study the morphology of the *M. glaucescens* and its geography.

Meanwhile, the nomenclature of *M. macranthus* commenced independently. Wallich (1830) first listed it as *Laurus macrantha* in his catalogue (No. 2587), based on collections from the Nilgiris by E. Noton [27]. Nees (1831) later transferred it to *Machilus*, publishing it as *M. macranthus* in *Plantae Asiaticae Rariores* describing it as having elliptic, acute leaves, glaucous and glabrous beneath, penninerved, with ample panicles, and pubescent branches which are dichotomously divided [25].

Further, Wight (1852) treated the species as *Machilus macrantha* (Nees), describing it with elliptic, acute leaves, glaucous and glabrous beneath, penninerved; large, pubescent panicles; ramuli divaricate bifid; and fruit globose, slightly depressed, approximately the size of a large currant. The species was reported from the ‘Nilgherries, on the northern and western slopes’ (Wight *Ic. t.* 1824) [19]. Dalzell and Gibson (1861), in *The Flora of Bombay*, recognised *M. macrantha* and *M. glaucescens* as separate species, referring to Wight’s figures of *M. macrantha* and *M. glaucescens* [28]. However, they were referring to Wight’s *Ic. Plantarum Indiae Orientalis* (1824 & 1825), with *M. macrantha* reported from “Parwar Ghaut, plentiful” and *M. glaucescens* from “The Ghauts”. Candolle (1864) additionally used the term excluded synonym (excl. syn.) for *M. glaucescens* to explicitly exclude Wight’s *M. glaucescens* from *M. macrantha* [29]*.*

Although the distribution of *M. glaucescens* (in Indo-Burma hotspots) had been confused with the Western Ghats, the name was not treated as a synonym until Beddome (1863), who combined the two species, adopted the name *M. macrantha*, and relegated *M. glaucescens* to synonymy [20] of *M. macranthus*. In the taxonomic revision of the genus *Machilus* in the ‘Indo-Burmese region’, both species were treated as varieties based on incomplete morphological observations [14].

Although *M. glaucescens* and *M. macranthu*s are taxonomically treated as varieties of the *M. macranthus*, their morphological differences, geographic distributions and distinct evolutionary lineages based on molecular studies raise doubts about current taxonomic status. Therefore, this study aims to clarify long-standing taxonomic uncertainties between *M. glaucescens* and *M. macranthus* by using: (a) a collection-based taxonomic approach, (b) morphometric analysis, (c) phylogenetic relationships, and (d) ecological niche modelling (ENM) to resolve the systematics and nomenclatural issues of the taxa. We demonstrate that the taxa are distinct species based on their morphology, genetic make-up, and distribution ranges.

## Materials and methods

### Ethics statement

Collections of *M. glaucescens* and *M. macranthus* were conducted in compliance with existing regulations for plants defined as non-commercial, as determined by regional government offices, in accordance with the Biological Diversity Act, 2002. In addition, these sample collections were performed in India with the written approval from the relevant communities, regional and governments, complying with Indian and International regulations for the collection of plant samples.

### Taxon sampling

**Fig 1:**
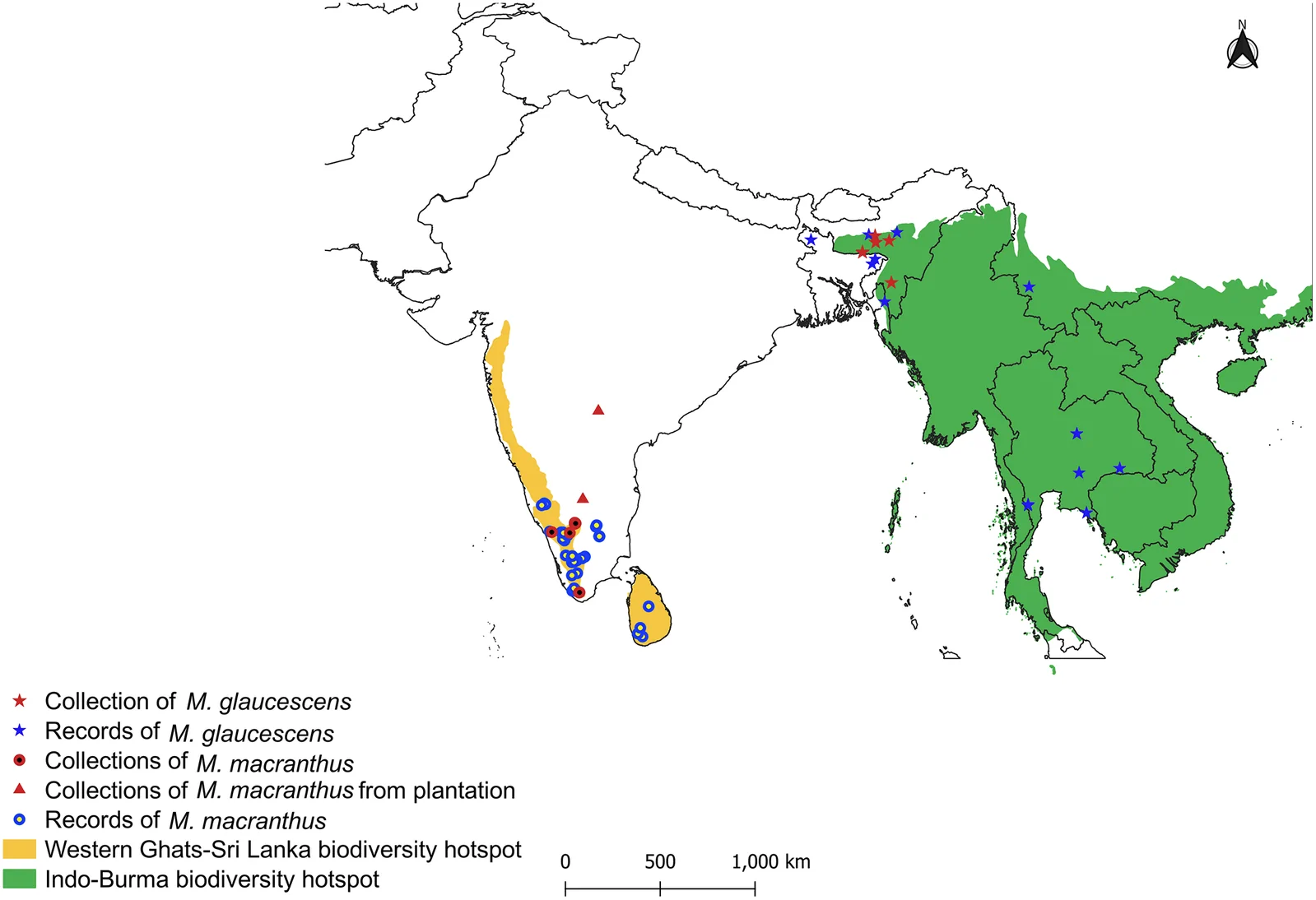
The distribution of *Machilus glaucescens* and *M. macranthus.* Locations of samples used for molecular analyses are indicated by red symbols (star, triangle, and dotted circle). The base map was obtained from the Natural Earth database (https://www.naturalearthdata.com/), public domain. Hotspot boundaries for Indo-Burma and Western Ghats-Sri Lanka were clipped from the global biodiversity hotspots dataset (version 2016.1; Mittermeier et al., 2011, *Global Biodiversity Conservation: The Critical Role of Hotspots*, in *Biodiversity Hotspots*, Springer), obtained from the Critical Ecosystem Partnership Fund (CEPF)/Conservation International (https://www.cepf.net/our-work/biodiversity-hotspots), used and modified (clipped to the study region) under CC BY-SA 4.0 (https://creativecommons.org/licenses/by-sa/4.0/). This element of the figure is licensed under CC BY-SA 4.0, distinct from the CC BY 4.0 license applied to the rest of this article. Map was prepared using QGIS v.3.28.

In the present study, we used plant collections of *M. glaucescens* from Assam, Meghalaya, and Mizoram of Northeast India and *M. macranthus* from Karnataka, Kerala and Tamil Nadu, of Western Ghats in addition to studies of collections deposited in herbaria (Fig 1). Fertile specimens (twigs with flowers and fruits) were collected for herbarium preparation. All voucher specimens were preserved following the standard herbarium method [30] and deposited in the ATREE herbarium. Leaf samples were cleaned with ethanol for surface sterilisation and stored at −20°C. Flowers and fruits were preserved in 70% alcohol for the morphological study. Types of both species available in the online database of Royal Botanic Gardens, Kew (K) were studied to confirm the identification of the collected plants.

### Morphological scoring

For the cluster analysis, 24 quantitative characters and four categorical characters were scored from six individuals for each species (S1 Table). Reproductive characters were measured from fully opened flowers, and fruit size from the matured fruits. Sizes were measured using an object micrometre and binocular microscope for the microscopic structures; otherwise, they were measured with a metric scale.

For linear measurement-based analysis, the following continuous characters were recorded: length and width of the largest and the smallest mature leaf, number of secondary veins, length of the petiole, internode, inflorescence, flower pedicel, length and width of outer and inner tepals, length of anther lobes in the 1st, 2nd, and 3rd whorls, length of anther filaments in the 1st, 2nd, and 3rd whorls, length of the glands, length and width of the ovary, length of the style-stigma, and staminodes. Categorical characters included presence/absence of hairs on leaves, equal/unequal leaf bases, presence/absence of hairs in inflorescences, and reflexed/spreading tepals of fruits (S1 Table).

### Morphometric analysis

Morphometric differences among species and individuals were assessed using Multiple Factor Analysis (MFA) on the combined dataset of 24 morphometric and four categorical variables. Analyses were performed using R package *FactoMineR* [31] and visualized with *Factoextra* [32]. Quantitative and categorical variables were first analysed separately using Principal Component Analyses (PCA) and Multiple Correspondence Analysis (MCA), respectively. Each dataset was normalized by dividing the loading scores of each dimension by the square root of the eigenvalue to prevent one data type from dominating the results. The normalized datasets were then combined in a final global PCA, to define the morphometric relationship between individuals and to calculate contribution of each data type to overall variation [32,33].

### DNA extraction, PCR amplifications and sequencing

Total genomic DNA was extracted from preserved leaf samples using a modified Cetyltrimethylammonium Bromide (CTAB) protocol [34]. Two nuclear regions were amplified: the Internal Transcribed Spacer (*ITS)* and *LEAFY* intron II [35,36]. The PCR program for *ITS* consisted of an initial denaturation at 95°C for 5 min; 35 cycles of 95°C for 45 s, 55°C for 1 min, and 72 °C for 1 min; followed by a final extension of 72°C for 10 min. Dimethyl Sulfoxide (DMSO) was added in all reactions to reduce issues caused by the secondary structures of GC-rich *ITS* regions [37,38]. The PCR program for *LEAFY* intron II included: 94°C for 2 min, 35cycles of 94°C for 30 s, 58°C for 2 min, and 72°C for 1 min; followed by a final extension of 72°C for 10 min. The sequencing was performed bidirectionally using Sanger sequencing at Barcode Biosciences, Bangalore, India.

### Alignment and phylogenetic analysis

Bidirectional sequences were manually examined using Chromas (http://technelysium.com.au/wp/chromas) and aligned using the Clustal W algorithm [39] with default settings in MEGA 7 [40]. Newly generated sequences were combined with the previously published data of the *Persea* group retrieved from GenBank for phylogenetic reconstruction (S2 Table). Following previous work [36], two species from the closely related genus *Litsea* and one species of *Lindera* were selected as outgroups. The dataset included 110 taxa (107 ingroup & 3 outgroup) and consisted of two nuclear regions (*ITS* and *LEAFY* intron II). Since both the genes are non- (protein) coding, the dataset was partitioned by gene. Maximum likelihood (ML) analysis was carried out on the IQ tree web server (http://iqtree.cibiv.univie.ac.at; [41]. IQ-TREE used inbuilt ModelFinder [42] to identify the best-fit substitution models for each partition (S3 Table). Bayesian inference (BI) was conducted in MrBayes 3.2 [43] with the appropriate best-fit models as suggested by PartitionFinder [44]; (S3 Table). Analysis was run for ten million generations, sampling every hundred generations, and terminated when the standard deviation of split frequencies fell below 0.001. Effective sample size (ESS) values were checked using the Tracer (v. 1.6) to confirm convergence (ESS > 200) for all parameters. Consensus trees were generated under the 50% majority-rule after discarding the first 25% as burn-in. Node support values >95 (Ultra-Fast Bootstrap, UFB) and >0.95 (Posterior Probability, PP) were considered strong [45,46].

### Ecological niche modelling

Species occurrence records for *M. glaucescens* and *M. macranthus* were sourced from a) field sampling, b) Global Biodiversity Information Facility (GBIF) records, and c) published literature. The samples collected from plantation areas (Fig 1) were excluded from the ecological niche modelling (ENM) data analysis. We examined herbarium images from GBIF to identify it as either *M. glaucescens* or *M. macranthus*, based on morphological traits and type specimens. Missing coordinates were georeferenced using Google Earth, and species records with missing coordinates and incomplete locality information were excluded. In total, 76 occurrence records were used (S4 Table): 21 for *M. glaucescens (*six from field collections and 15 obtained from GBIF) and 55 for *M. macranthus* (10 from field collections, 19 obtained from GBIF and 26 from published literature).

Ecological niche models were generated using 19 bioclimatic variables [annual mean temperature (BIO1), mean diurnal temperature range (BIO2), isothermality (BIO3), temperature seasonality (BIO4), maximum temperature of the warmest month (BIO5), minimum temperature of the coldest month (BIO6), annual temperature range (BIO7), mean temperatures of the wettest (BIO8), driest (BIO9), warmest (BIO10), and coldest (BIO11) quarters, as well as precipitation-related variables including annual precipitation (BIO12), precipitation of the wettest (BIO13) and driest (BIO14) months, precipitation seasonality (BIO15), and precipitation of the wettest (BIO16), driest (BIO17), warmest (BIO18), and coldest (BIO19) quarters] and the elevation data from the WorldClim database v2.0 (http://www.worldclim.org/) with a spatial resolution of 1 km^2^ (30 arc seconds). After performing a correlation analysis to reduce multicollinearity, eight variables (BIO1, BIO2, BIO3, BIO12, BIO14, BIO15, BIO19, and elevation) were retained for niche modelling, while the remaining variables (BIO4–BIO11, BIO13, and BIO16–BIO18) were excluded.

To develop the ecological niche model, we used the Maximum Entropy (MaxEnt) method [47,48]. For *M. macranthus*, models were constructed using default settings. For *M. glaucescens*, which had a smaller number of occurrence records, model complexity was reduced by restricting feature classes to linear, quadratic, and hinge features, following recommendations by Merow et al., 2013 [49]. Model performance was evaluated using 10-fold cross-validation. The number of background points was set to 10,000, and the maximum number of iterations was increased to 1,000, with a convergence threshold of 1 × 10^−6^. Jackknife tests were performed to assess the relative importance of predictor variables. The “10th percentile training presence” threshold was selected to reduce the influence of potential errors and spatial uncertainty in occurrence records derived from multiple sources. Model performance was evaluated using the AUC (area under the curve) score.

Predicted distributions were converted into binary presence-absence maps using the 10th percentile training presence threshold. Habitats below the threshold were considered absent, and those above were considered present. Habitat suitability maps for both species were generated using the raster calculator tool in QGIS (v 3.28) and visualized to identify areas of suitable and unsuitable habitat under current environmental conditions.

Habitat overlap between the two species was quantified using the PCA-env method [50]. The first two environmental axes (PC1 and PC2) were divided into a 100 × 100 grid. For each species, occurrence density in environmental space was estimated using a smoothing approach and plotted on a two-dimensional surface for each species. Finally, overlap between the species was measured using Schoener’s D, a value that goes from 0 (no overlap) to 1 (complete overlap). All analyses were done in R using the *ecospat* package.

## Results

### Morphology of *M. glaucescens* and *M. macranthus*

Morphological examination of the collected specimens confirmed that the samples represented two distinct species, *M. glaucescens* and *M. macranthus*, both consistent with the genus characters of *Machilus*. Comparison with protologues [25] and type specimens (https://plants.jstor.org/collection/TYPSPE) available at Royal Botanic Gardens, Kew (K) corroborated this identification.

The two species shared genus characteristics but differed in terms of several morphological characteristics. *M. glaucescens* was distinguished by leaf shape; oblong-lanceolate, hairy on both surfaces, with acute apex and base; panicles reddish-brown and tomentose. In contrast, *M. macranthus* was identified based on- leaves glabrous; panicles large, pubescent, and divaricate.

**Fig 2:**
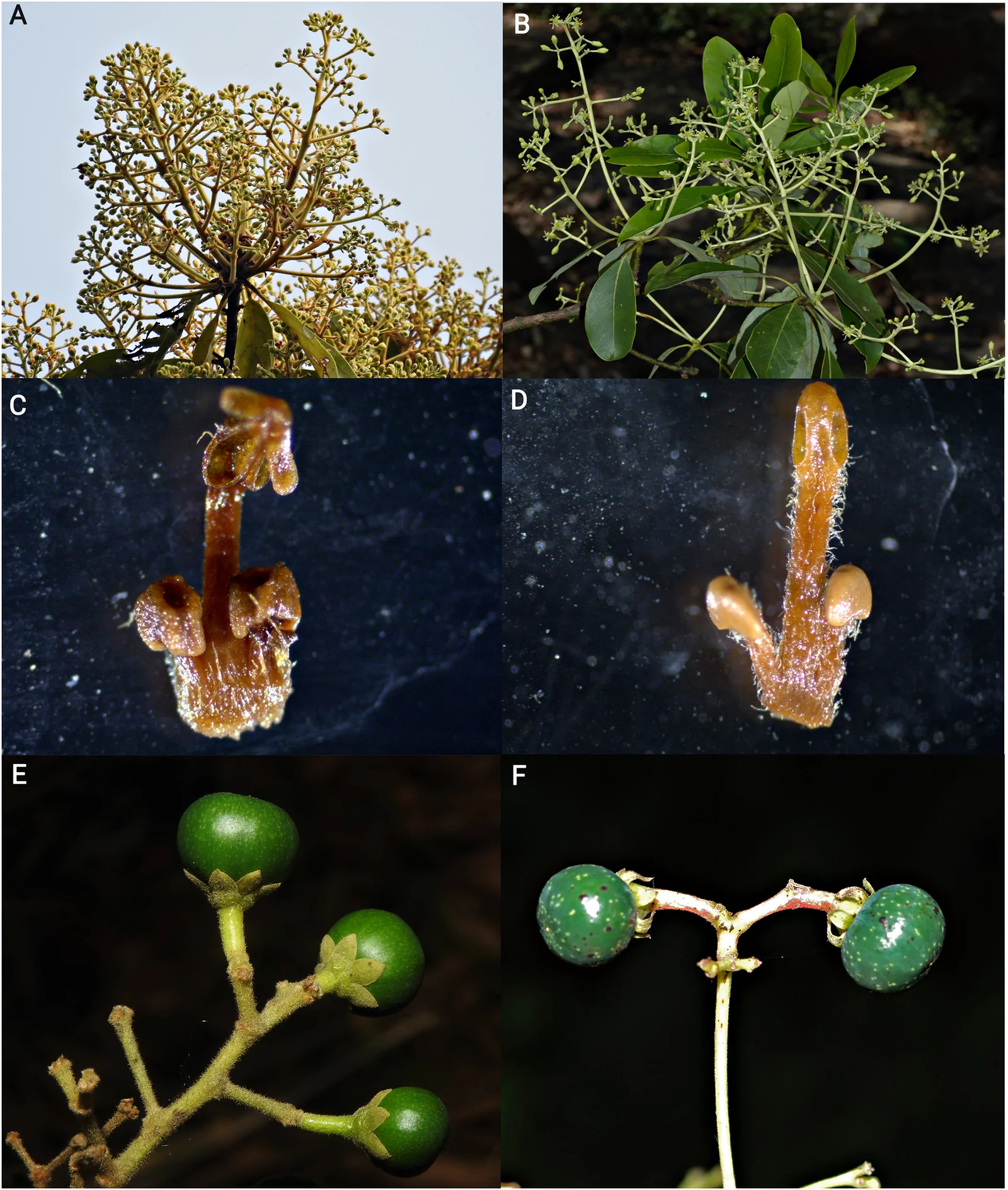
Distinguishing morphological characteristics of *M. glaucescens* and *M. macranthus.* A&B. Panicles with dense inflorescences and reddish-brown hairs in *M. glaucescens* (A) vs. loosely packed inflorescences in *M. macranthus* (B). C&D. Filaments of stamens glabrous in *M. glaucescens* (C) vs. tomentose in *M. macranthus* (D). E&F. Tepals spreading in fruits of *M. glaucescens* (E) vs. reflexed in *M. macranthus* (F) (Photos by Ganesan R. & Nabasmita M.).

Additional to these morphological features drawn from the protologues, other distinguishing characters between the *M. glaucescens* and *M. macranthus* include base of the leaves equal vs. unequal, leaves hairy vs. glabrous, venation foveolate vs. non-foveolate, panicles dense vs. loosely packed, and tepals in fruits spreading vs. reflexed. A summary of the observed differences from plant collections across their distributional range is presented in Table 1 and Fig 2.

### Morphometry of *M. glaucescens* and *M. macranthus*

**Fig 3:**
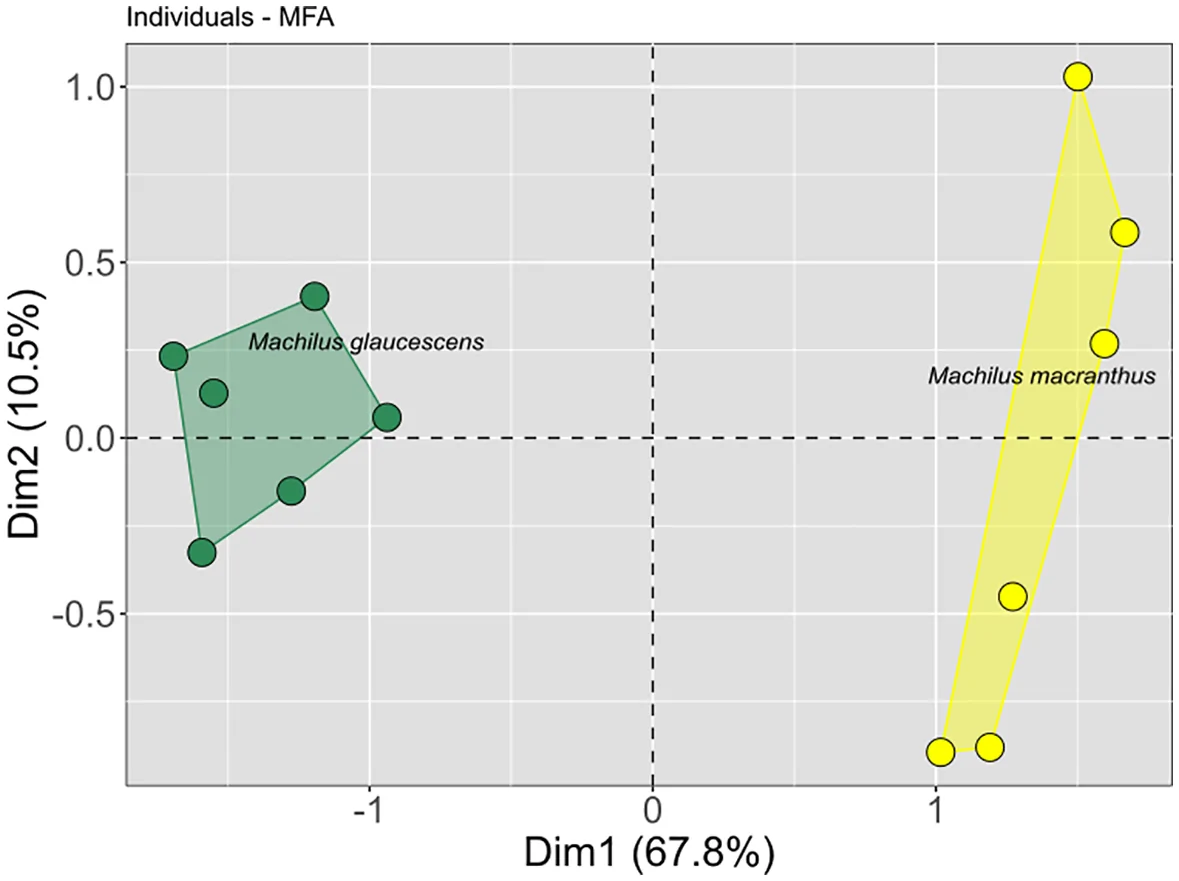
Multiple Factor Analysis (MFA) scatter plot of the morpho-spatial relationships between M. glaucescens and M. macranthus, showing two distinct clusters.

The Multiple Factor Analysis (MFA) based on 24 quantitative and four categorical morphological characters distinctly separated the specimens into two distinct clusters, corresponding to *M. glaucescens* and *M. macranthus* (Fig 3). This result indicates clear morphological differentiation between the two species.

The first dimension (Dim) of the MFA explained 67.8% and dimension 2 accounted for 10.5% of the total variation within the morphological trait dataset of *M. glaucescens* and *M. macranthus.* Both morphometric and categorical traits contributed almost equally to Dim-1 (approximately 50% each). In contrast, Dim-2 was explained primarily by morphometric traits (96%), with only a small contribution from categorical traits (3%) of the data. Similarly, morphometric traits accounted for most of the variation in Dim-3 (84%) and 4 (100%), whereas categorical traits contributed 15% and negligible variation, respectively (S1 Fig).

Overall, morphological differences between the species were driven primarily by continuous traits such as leaf size, inflorescence length, and floral measurements, while categorical traits (e.g., equal vs. unequal leaf bases, reflexed vs. spreading tepals) contributed less to species separation.

### Phylogeny of *M. glaucescens* and *M. macranthus*

The aligned ITS dataset (556 bp) contained 390 variable sites, of which 124 were parsimony informative. The *LEAFY* dataset (736 bp) included 360 variable sites and 208 parsimony-informative sites. The concatenated matrix (1292 bp) had 750 variable sites and 332 parsimony-informative sites. The genus *Machilus* was retrieved as monophyletic with strong support (PP = 1, UFB = 100), sister to the genus *Persea* with strong support (PP = 0.96, UFB = 94) within the *Persea* group.

**Fig 4:**
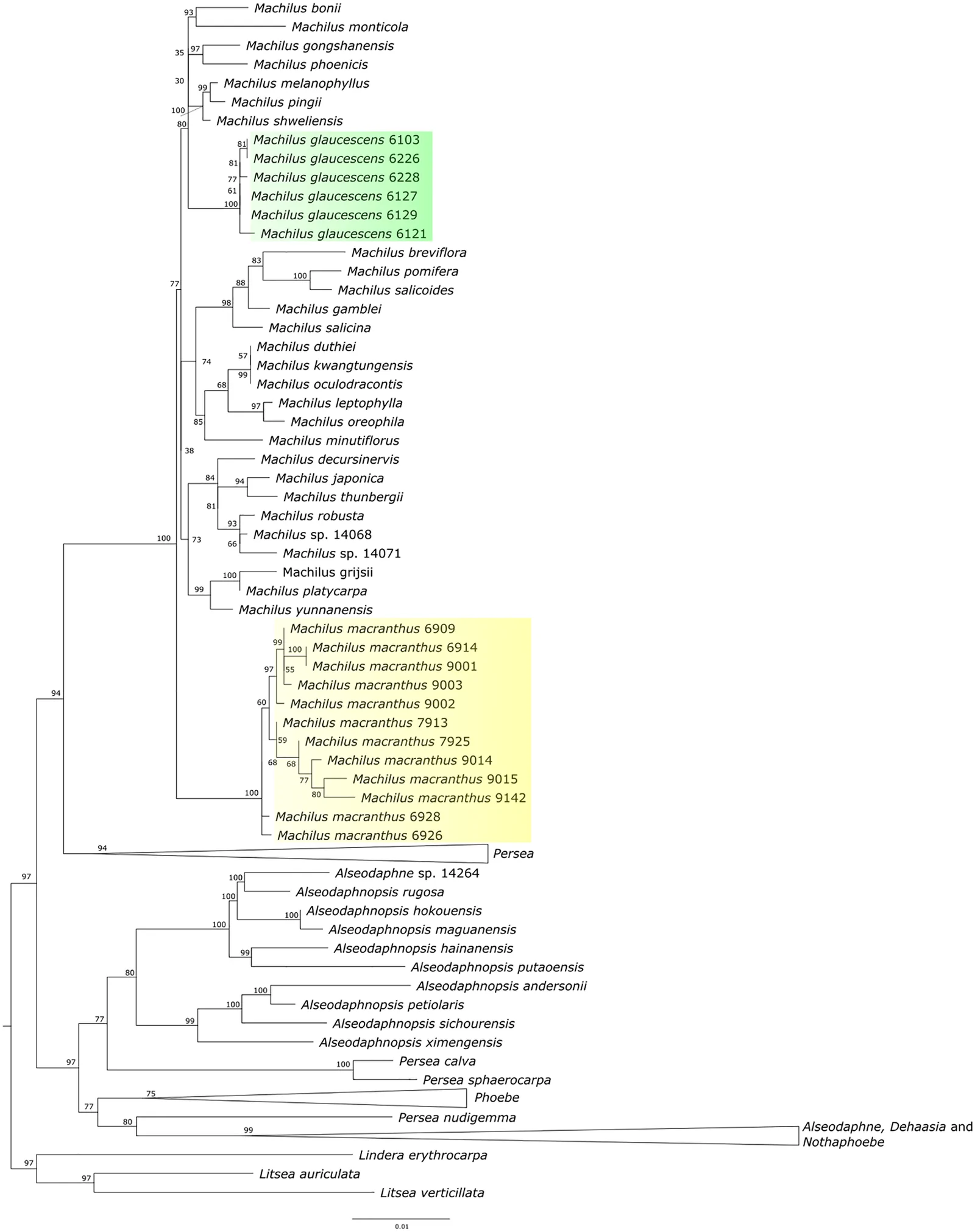
Maximum Likelihood (ML) tree based on nr*ITS* and *LEAFY* sequences showing the phylogenetic positions of *M. glaucescens* and *M. macranthus* within the Persea group. Numbers at the nodes indicate UltraFast Bootstrap (UFB) values. The complete ML tree is provided in the supplementary materials (S3 Fig).

All newly generated sequences of *M. glaucescens* and *M. macranthus* were nested within the monophyletic genus *Machilus* of the *Persea* group (Fig 4). In the Maximum Likelihood tree, *M. glaucescens* was nested within a clade containing *M. bonii*, *M. monticola*, *M. gongshanensis*, *M. phoenicis*, *M. melanophyllus*, *M. pingii*, and *M. shweliensis* from south-central China and Myanmar, with moderate support (UFB = 80). *Machilus macranthus* formed an early-diverging lineage, sister to all other *Machilus* species, also with moderate support (UFB = 77, Fig 4). In the Bayesian Inference analysis, samples of both *M. glaucescens* and *M. macranthus* were monophyletic, however their relationships with respect to other species were unresolved (S2 Fig.).

### Ecological niche and niche overlap of *M. glaucescens* and *M. macranthus*

Observations in the field and herbarium specimens confirm the distribution of *M. glaucescens* in the Indo-Burma biodiversity hotspot and *M. macranthus* in the Eastern Ghats and Western Ghats-Sri Lanka hotspots of biodiversity. From our examinations, we determined that 22 out of 133 herbarium sheets from the GBIF were wrongly assigned. Our examinations of herbarium collections from GBIF further indicate that *M. macranthus* is misidentified more frequently than *M. glaucescens* (S5 Table).

**Fig 5:**
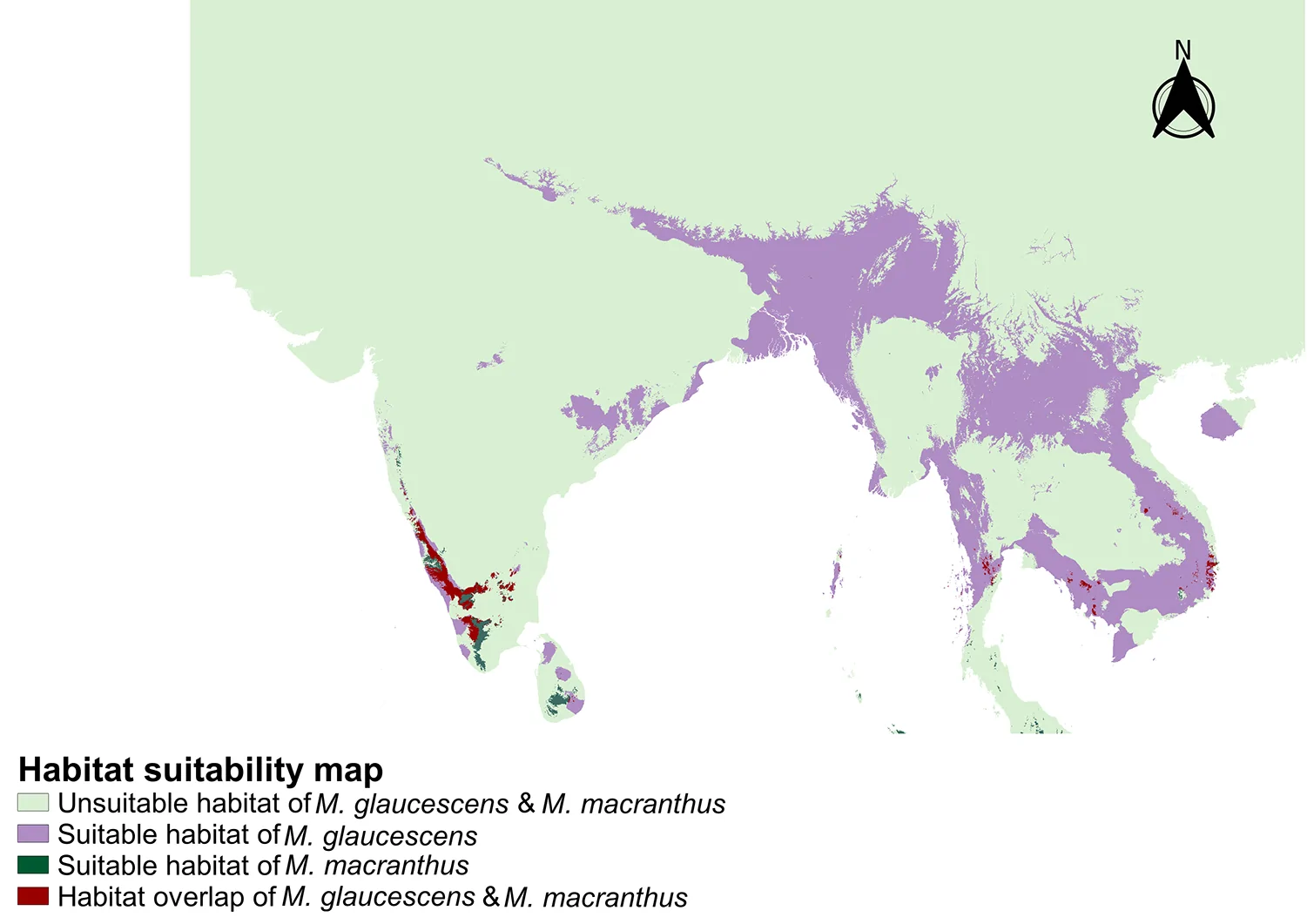
Projected habitat suitability and habitat overlap of *M. glaucescens* and *M. macranthus.*

MaxEnt models for both species showed high predictive performance, with area under the curve (AUC) values exceeding 0.90 (S5 Fig). Although *M. glaucescens* is currently limited to the Indo-Burma hotspot, potential suitable habitats for this species were projected in northern and central Western Ghats, whereas *M. macranthus* is currently restricted to the Eastern and Western Ghats- Sri Lanka hotspots, and ENM predicted parts of Indo-Burma hotspot as suitable habitat (Fig 5, S4 Fig). Niche overlap was minimal: Schoener’s D index was low (D = 0.447), indicating largely distinct climatic preferences for the two species.

## Discussion

The family Lauraceae has long been recognized for its complex taxonomy and incorrect identifications [1,51,52]. The taxonomic routes of *M. glaucescens* and *M. macranthus* exemplify this trend, as both species have undergone repeated shifts in generic and specific status owing to inadequate examination of morphology and distribution. Although *M. glaucescens* and *M. macranthus* exhibit clear morphological and distributional distinctions, the absence of comprehensive collection-based research has perpetuated the confusion regarding their taxonomic identifications. The recent classification of *M. glaucescens* and *M. macranthus* [14] to infraspecific varieties fails to accurately depict their true taxonomic status, and the study overlooked geographical distribution in addition to key morphological differences and phylogenetic relationships. The morphological differences between *M*. *glaucescens* and *M. macranthus,* listed in Table 1, demonstrate that they should be recognised as two distinct species rather than varieties. This argument is further supported by collection-based taxonomy, molecular distinctness, morphometric analysis and ecological niche modelling.

Our morphometric analyses, integrating both quantitative and qualitative traits through Multiple Factor Analysis (MFA), enable the simultaneous analysis of different types of data while ensuring that each data group contributes equally to the overall variation [33]. Our results further demonstrate that quantitative traits are particularly valuable in resolving species boundaries within Lauraceae, a family in which such approaches have rarely been applied, largely due to the limited availability of collections in flowering or fruiting [52]. This pattern is consistent with findings in other plant groups, including Alliaceae, Juglandaceae, Ephedraceae, and Clusiaceae, where morphometric approaches have helped clarify species limits [53–56]. Nevertheless, while quantitative characters such as size of the inflorescence, size of tepals and anthers contributed more to the analyses, we emphasize that qualitative traits remain equally important for the identification of species. The qualitative characters of *M. glaucescens* and *M. macranthus* such as leaves -hairy vs. glabrous, leaf base- equal vs. unequal, venation -foveolate vs. non-foveolate, panicles -dense vs. loosely packed, and tepals in fruits spreading vs. reflexed are important to distinguish both species. The two distinct clusters of these species further highlight their clear morphological differentiation. Within the species, *M. macranthus* exhibits greater morphological variation than *M. glaucescens*, a pattern that is also reflected in the clustering observed in the MFA analysis.

Likewise, the molecular phylogenetic relationship contradicts [14,20] the idea of a close relationship between *M. glaucescens* and *M. macranthus.* The placement of *M. glaucescens* alongside other species from south-central China and Myanmar reflects biogeographic proximity, as north-eastern India shares affinities with the Indo-Burma and Eastern Himalaya hotspots, which differ sharply from those of the Eastern and Western Ghats biodiversity hotspots of India. The single known species of the genus *Machilus* (i.e., *M. macranthus*) in Western Ghats and Eastern Ghats could also be the relic of a former continuous distribution [57], a pattern similarly observed in few other taxa [58–60]. Notably, *M. glaucescens* exhibits a closer phylogenetic affinity to *Machilus* species from the Indo-Burma region than to *M. macranthus*. The non-sister relationship between *M. glaucescens* and *M. macranthus* further indicates their distinct evolutionary history, with *M. macranthus* representing an earlier-diverging lineage.

Our study suggests that misidentifications have resulted in wrong distribution records for both the species in global databases such as GBIF (https://www.gbif.org/) and POWO (https://powo.science.kew.org/). This pattern has also been reported in several earlier studies, which showed that taxonomic misidentifications can distort our understanding of biodiversity [61–63]. From this study, it is established that the *M. glaucescens* is distributed in Indo-Burma and *M. macranthus* is distributed in the Eastern Ghats and Western Ghat-Sri Lanka biodiversity hotspots. Ecological niche modelling results further support the existence of distinct climatic niches for both species. We examined 133 herbarium specimens from GBIF and found 22 were misidentified. Furthermore, *M. macranthus* appears to be misidentified more frequently than *M. glaucescens*. Morphological traits such as an equal versus unequal leaf base, spreading versus reflexed tepals, and dense versus loosely arranged panicles are particularly useful for distinguishing online herbarium specimens.

These long-standing taxonomic ambiguities have obscured the true distributional ranges of *M. glaucescens* and *M. macranthus*, with important implications for conservation planning. Accurately delimiting these species is therefore crucial not only from a taxonomic and evolutionary perspective but also to understand their actual distributions and inform effective conservation strategies. The integrative taxonomic approach used in this study iterates the importance of the approach that should be used to resolve the existing uncertainties in the biogeography of many plant taxa in India and globally.

## Conclusion

Through this study, using an integrative taxonomic approach (collection-based taxonomy, morphometric analysis, phylogenetics and ecological niche modelling), we provided evidence to resolve the long-standing taxonomic confusion between *M. glaucescens* (Nees) Wight and *M. macranthus* (Nees). We consider these taxa, which were previously treated as two infraspecific varieties, *M. macranthus* var. *glaucescens* (Nees) Chakrab., Ghoshal, Anand Kumar & V. K. Rawat and *M. macranthus* var. *macranthus,* two distinct species status as *M. glaucescens* (Nees) Wight and *M. macranthus* (Nees). This reclassification is supported by robust evidence from morphology, phylogeny and ecological niche distribution modelling.

## Supporting information

S1 Table Morphological scoring of *M. glaucescens* and *M. macranthus.* The details of quantitative and qualitative morphological traits of both species.
(XLSX)

S2 Table Voucher information and GenBank accessions for ITS and LFY sequences for species examined in this study. The newly generated sequences in this study are presented in bold, and “-” refers to the unavailability of the sequence. GenBank accessions beginning with AF are from [43], with AY are from Li *et al.* (unpublished), with FJ from [64], with FM from [1], with HQ from [36], with MG from [65], with MN from [66], with MW from [67], with PV from [68]. Sequences for species shown in bold were generated in this study.
(DOCX)

S3 Table Nucleotide substitution model. The best-fit nucleotide substitution model of the two markers for both BI and ML analyses.
(DOCX)

S4 Table Occurrence data of *M. glaucescens* and *M. macranthus* were compiled from secondary sources and field survey records. (XLSX)

S5 Table Details of the examined herbarium specimens obtained from online sources. (XLSX)

S1 Fig Bar graphs depicting the percentage contribution of A) categorical and morphometric characters in the Dim-1 B) morphometric and categorical characters in the Dim-2 C) morphometric and categorical characters in the Dim-3 D) morphometric and categorical characters in the Dim-4. The dashed red color line represents the expected average contribution if all data types contributed equally.
(DOCX)

S2 Fig The complete Bayesian tree based on nr*ITS* and *LEAFY* sequences showing the phylogenetic positions of *M. glaucescens* and *M. macranthus* within the Persea group. Numbers at the nodes indicate Posterior probability (PP) values.
(DOCX)

S3 Fig The complete Maximum Likelihood tree based on nr*ITS* and *LEAFY* sequences showing the phylogenetic positions of *M. glaucescens* and *M. macranthus* within the Persea group. Numbers at the nodes indicate UltraFast Bootstrap (UFB) values.
(DOCX)

S4 Fig (A) Predicted habitat suitability of *Machilus glaucescens* (B) Predicted habitat suitability of *Machilus macranthus.* (DOCX)

S5 Fig Receiver Operating Characteristic (ROC) curve generated from one of the 10-fold cross-validation runs of the MaxEnt model for A) *M. glaucescens* and B) *M. macranthus.* (DOCX)
