# Supplementary material for: Two regions, two lineages: Untangling two centuries of confusion between Machilus glaucescens and M. macranthus (Lauraceae) through integrative taxonomy
Source: PLoS One. 2026 Sep 9;21(9):e0357315. doi: 10.1371/journal.pone.0357315 (PMC13557376; doi:10.1371/journal.pone.0357315)
Supplement: Supplementary file 1 [file pone.0357315.s002.docx]

**Supplementary Information 2**

Table 1. **Voucher information and GenBank accessions for ITS and LFY sequences for**

**species examined in this study**. The newly generated sequences in this study are presented in bold, and “-” refers to the unavailability of the sequence. GenBank accessions beginning with AF are from [43], with AY are from Li *et al.* (unpublished), with FJ from [64], with FM from [1], with HQ from [36], with MG from [65], with MN from [66], with MW from [67], with PV from [68]. Sequences for species shown in bold were generated in this study.

| **Taxon** | **Voucher** | **Locality** | **ITS** | **LEAFY** | **Reference** |
| --- | --- | --- | --- | --- | --- |
| Alseodaphne gracilis | Li L.et al. GLQ23 (HITBC) | China, Yunnan | MG188581 | MG188659 |  |
| Alseodaphne huanglianshanensis | Li L. 20080006 (HITBC) | China, Yunnan | HQ697182 | HQ697007 |  |
| Alseodaphne semecarpifolia | Arifiani DA658 (BO) | Indonesi,Java | HQ697184 | HQ697013 |  |
| Alseodaphne sp. W14264 | van der Werff & Nguyen 14264 (MO) | Vietnam, Tuyen Quang | FM957797 | HQ697016 |  |
| Alseodaphne sp. W17084 | van der Werff et al., 17084 (MO) | Vietnam, Lang Son | FM957798 | HQ697018 |  |
| Alseodaphnopsis andersonii | Li J. & Li L. 20070074 (HITBC) | China, Yunnan | FM957793 | HQ697002 |  |
| Alseodaphnopsis hainanensis | Li L. & Wang Z. H. 20070317 (HITBC) | China, Hainan | FJ755440 | HQ697005 |  |
| Alseodaphnopsis hokouensis | Bing Liu 2358 | China, Yunnan | MW826237 | MW849583 |  |
| Alseodaphnopsis maguanensis | Li L. et al. GLQ45 (HITBC) | China, Yunnan | MN906900 | MN906896 |  |
| Alseodaphnopsis petiolaris | Chen J. Q. 07003 (HITBC) | China, Yunnan | FM957796 | HQ697008 |  |
| Alseodaphnopsis putaoensis | Li L. & Ma H. MM254 (HTBC) | Myanmar, Kachin | MN906902 | MN906898 |  |
| Alseodaphnopsis rugosa | Li L. & Wang Z. H. 20070369 (HITBC) | China, Hainan | HQ697183 | HQ697011 |  |
| Alseodaphnopsis sichourensis | Song Y. 33225 (HITBC) | China, Yunnan | MG188597 | MG188626 |  |
| Alseodaphnopsis ximengensis | Li J. W. 1235 (HITBC) | China, Yunnan | MG188591 | MG188599 |  |
| Dehaasia hainanensis | Li L. & Wang Z. H. 20070373 (HITBC) | China, Hainan | FJ719308 | HQ697025 |  |
| Dehaasia incrassata | X.H.Jin IN004 | Indonesia | MW826238 | MW849585 |  |
| Dehaasia sp. A34 | Arifiani & van der Werff 34 (MO) | Indonesi, Java | FM957805 | HQ697031 |  |
| Dehaasia sp. L20070187 | Li L. 20070187 (HITBC) | China, Yunnan | HQ697187 | HQ697034 |  |
| Machilus bonii | Bing Liu 2133 | China, Yunnan | MW826241 | MW849588 |  |
| Machilus breviflora | Chen J. Q. et al., 2006013(HITBC) | China, Guangdong | FJ755434 | HQ697041 |  |
| Machilus decursinervis | Li J. 2002195 (HITBC) | China, Guangxi | AY934893 | HQ697044 |  |
| Machilus duthiei | Zhong J. S. 2006094 (HITBC) | China, Yunnan | FJ755425 | HQ697054 |  |
| Machilus gamblei | Chen J. Q. et al., 2006001 (HITBC) | China, Guangdong | FJ755422 | HQ697037 |  |
| **Machilus glaucescens** | **ATREE6103** | **Karbi-Anglong, Assam** | **PZ538614** | **PZ535131** |  |
| **Machilus glaucescens** | **ATREE6121** | **Mizoram University campus, Mizoram** | **PZ538615** | **PZ535132** |  |
| **Machilus glaucescens** | **ATREE6127** | **Nongkalong, Meghalaya** | **PZ538616** | **PZ535133** |  |
| **Machilus glaucescens** | **ATREE6129** | **Ranikor, Meghalaya** | **PZ538617** | **PZ535134** |  |
| **Machilus glaucescens** | **ATREE6226** | **Umling, Meghalaya** | **PZ538618** | **PZ535135** |  |
| **Machilus glaucescens** | **ATREE6228** | **Umsning Meghalaya** | **PZ538619** | **PZ535136** |  |
| Machilus gongshanensis | Chen J. Q. 07002 (HITBC) | China, Yunnan | FJ755416 | HQ697046 |  |
| Machilus grijsii | Chen J. Q. et al., 2006028 (HITBC) | China, Guangdong | FJ755420 | HQ697048 |  |
| Machilus japonica | Kim C. K. s.n. (HBG) | Korea, Cheju | AY934891 | HQ697050 |  |
| Machilus kwangtungensis | Chen J. Q. et al., 2006027 (HITBC) | China, Guangdong | FJ755424 | HQ697051 |  |
| Machilus leptophylla | Li J. & Li L. 20070190 (HITBC) | China, Zhejiang | FJ755430 | HQ697053 |  |
| Machilus melanophyllus | Bing Liu 1950 | China, Yunnan | MW826243 | MW849590 |  |
| Machilus minutiflorus | Chen J. Q. et al., 2005038 (HITBC) | China, Yunnan | HQ697208 | HQ697147 |  |
| **Machilus macranthus** | **ATREE6909** | **BRT, Karnataka** | **PZ538620** | **PZ535137** |  |
| **Machilus macranthus** | **ATREE6914** | **BRT, Karnataka** | **PZ538621** | **PZ535138** |  |
| **Machilus macranthus** | **ATREE6926** | **Gopanpally, Telangana** | **PZ538622** | **PZ535139** |  |
| **Machilus macranthus** | **ATREE6928** | **Nalumukku, KMTR, Tamil Nadu** | **PZ538623** | **PZ535140** |  |
| **Machilus macranthus** | **ATREE7913** | **Wayanad, Kerala** | **PZ538624** | **PZ535141** |  |
| **Machilus macranthus** | **ATREE7925** | **BRT, Karnataka** | **PZ538625** | **PZ535142** |  |
| **Machilus macranthus** | **ATREE9001** | **BRT, Karnataka** | **PZ538626** | **PZ535143** |  |
| **Machilus macranthus** | **ATREE9002** | **BRT, Karnataka** | **PZ538627** | **PZ535144** |  |
| **Machilus macranthus** | **ATREE9003** | **BRT, Karnataka** | **PZ538628** | **PZ535145** |  |
| **Machilus macranthus** | **ATREE9014** | **FRLHT campus, Karnataka** | **PZ538629** | **PZ535146** |  |
| **Machilus macranthus** | **ATREE9015** | **Kakachi, KMTR, Tamil Nadu** | **PZ538630** | **PZ535147** |  |
| **Machilus macranthus** | **ATREE9142** | **Nilgiri, Tamil Nadu** | **PZ538631** | **PZ535148** |  |
| Machilus monticola | Li L. & Wang Z. H. 20070323 (HITBC) | China, Hainan | FJ755418 | HQ697056 |  |
| Machilus oculodracontis | Chen J. Q. et al., 2006037 (HITBC) | China, Guangdong | HQ697188 | HQ697059 |  |
| Machilus oreophila | Chen J. Q. et al., 2006067 (HITBC) | China, Guangdong | FJ755423 | HQ697063 |  |
| Machilus phoenicis | Chen J. Q. et al., 2006009 (HITBC) | China, Guangdong | FJ755413 | HQ697064 |  |
| Machilus pingii | Li L. 20070263(HITBC) | China, Sichuan | HQ697189 | HQ697065 |  |
| Machilus platycarpa | Chen J. Q. et al., 2006073 (HITBC) | China, Guangdong | FJ755421 | HQ697067 |  |
| Machilus pomifera | Chen J. Q. et al., 2006064 (HITBC) | China, Guangdong | FJ755432 | HQ697069 |  |
| Machilus robusta. | Li J. 2002116 (HITBC) | China, Guangxi | FJ755426 | HQ697071 |  |
| Machilus salicina | Chen J. Q. et al., 2005001 (HITBC) | China, Yunnan | FJ755428 | HQ697073 |  |
| Machilus salicoides | Chen J. Q. et al., 2006090 (HITBC) | China, Guangdong | FJ755433 | HQ697074 |  |
| Machilus shweliensis | Li J. 2002087 (HITBC) | China, Guangxi | FJ755414 | HQ697075 |  |
| Machilus sp. W14068 | van der Werff et al., 14068 (MO) | Vietnam, Vinh Phu | FM957812 | HQ697077 |  |
| Machilus sp. W14071 | van der Werff et al., 14071 (MO) | Vietnam, Vinh Phu | FM957813 | HQ697079 |  |
| Machilus thunbergii | Rohwer s.n. (HBG) | Germany, Hamburg | HQ697190 | HQ697081 |  |
| Machilus yunnanensis | Zhong J. S. 2006093 (HITBC) | China, Yunnan | FJ755415 | HQ697083 |  |
| Nothaphoebe gigaphylla | Arifiani DA657 (BO) | Indonesia, Java | HQ697181 | HQ697003 |  |
| Nothaphoebe umbelliflora | Arifiani DA495 (BO) | Indonesia, Java | HQ697191 | HQ697088 |  |
| Persea alba | Borgo & Britez 2165 (MO) | Brazil, Paraná | HQ697192 | HQ697089 |  |
| Persea americana | Li J. 2002001 (HITBC) | China, Yunnan | AF272322 | HQ697091 |  |
| Persea areolatocostae | Quizhpe 208 (MO) | Ecuador, Zamora-Chinchipe | HQ697193 | HQ697095 |  |
| Persea aurata | Folli 4089 (MO) | Brazil, Espirito Santo | HQ697194 | HQ697097 |  |
| Persea barbujana | Rohwer s.n. (HBG) | Spain, Canary Islands | AY934889 | HQ697021 |  |
| Persea borbonia | Kirchner s.n. (HBG) | Germany, Bochum | AY934901 | HQ697098 |  |
| Persea caerulea | Linares 5213 (MO) | Honduras, Depto Francisco Morazán Mipo | FJ755436 | HQ697099 |  |
| Persea haenkeana | Fuentes 7370 (MO) | Bolivia, La Paz Bautista Saavedra | HQ697195 | HQ697100 |  |
| Persea indica | Rohwer s.n. (HBG) | Germany, Hamburg | AY934902 | HQ697101 |  |
| Persea lingue | Aedu 7242 (MO) | Chile, La Araucania | HQ697196 | HQ697102 |  |
| Persea major | Araujo 216 (MO) | Brazil, M G Bias Fertes | HQ697197 | HQ697103 |  |
| Persea nudigemma | van der Werff et al., 19209 (MO) | Ecuador, Zamora-Chinchipe | FM957828 | HQ697105 |  |
| Persea palustris | Miller et al., 9018 (MO) | USA, Florida, Leon | FM957831 | HQ697107 |  |
| Persea sp. B21834 | Beck 21834 | Bolivia (HBG) | HQ697198 | HQ697109 |  |
| Persea sp. V25232 | Vásquez & Ortiz-Gentry 25232 (MO) | Peru, Loreto, Maynas | FM957836 | HQ697110 |  |
| Persea sp. W14857 | van der Werff et al., 14857 (MO) | Peru, Amazonas | FM957833 | HQ697111 |  |
| Persea sp. W19517 | van der Werff et al., 19517 (MO) | Ecuador, Zamora-Chinchipe | FM957834 | HQ697113 |  |
| Persea calva | van der Werff 21874 (MO) | Ecuador | FM957835 | HQ697116 |  |
| Persea sphaerocarpa | van der Werff et al., 17889 (MO) | Peru, Pasco, Oxapampa | FM957837 | HQ697117 |  |
| Persea splendens | Fonseca 2568 (MO) | Brazil, Goiás Municipio Campos Belos | HQ697199 | HQ697119 |  |
| Persea steyermarkii | Monterrosa 408 (MO) | El Salvador, Dept. Santa Ana | HQ697200 | HQ697120 |  |
| Persea weberbaueri | van der Werff 21626 (MO) | Ecuador | FM957842 | HQ697122 |  |
| Phoebe arnottii | ATREE7334 | Kodayar, Tamil Nadu | PV962255 | PV959543 |  |
| Phoebe angustifolia | Li L. 20070058 (HITBC) | China, Yunnan | HQ697201 | HQ697124 |  |
| Phoebe cavaleriei | Bing Liu 2215 | China, Yunnan | MW826245 | - |  |
| Phoebe chekiangensis | Li J. & Li L. 20070188 (HITBC) | China, Zhejiang | FJ755407 | HQ697127 |  |
| Phoebe cuneata | Arifiani 40 (MO) | Indonesia | HQ697202 | HQ697129 |  |
| Phoebe elliptica | Samsuri & Gwee SING2004-28 (SBG) | Singapore | HQ697203 | HQ697131 |  |
| Phoebe formosana | Rohwer 156 (MJG) | Germany, Bonn | HQ697205 | HQ697135 |  |
| Phoebe hungmaoensis | Li L. & Wang Z. H. 20070306 (HITBC) | China, Hainan | HQ697206 | HQ697137 |  |
| Phoebe jinpingensis | Bing Liu 1477 | China, Yunnan | MW826231 | MW849584 |  |
| Phoebe lanceolata | Chen J. Q. et al., 2006093 (HITBC) | China, Guangdong | FJ755410 | HQ697141 |  |
| Phoebe macrocarpa | Li J. 2002207 (HITBC) | China, Guangxi | FJ755408 | HQ697142 |  |
| Phoebe megacalyx | Li J. & Li L. 20070026 (HITBC) | China, Yunnan | HQ697207 | HQ697144 |  |
| Phoebe neurantha | Li J. & Li L. 20070214 (HITBC) | China, Zhejiang | HQ697209 | HQ697151 |  |
| Phoebe neurantha var. brevifolia | Bing Liu 2270 | China, Yunnan | MW826253 | MW849598 |  |
| Phoebe puwenensis | Chen J. Q. et al., 2006065 (HITBC) | China, Guangdong | HQ697210 | HQ697152 |  |
| Phoebe sheareri | Bing Liu 2224 | China, Hubei | MW826256 | MW849601 |  |
| Phoebe tavoyana | Bing Liu 1822 | China, Hainan | MW826257 | - |  |
| Phoebe sp. A49 | Arifiani 49 (MO) | Indonesia | HQ697211 | HQ697158 |  |
| Phoebe sp. L20070260 (Not. cavaleriei (Lévl.)Yang) | Li L. 20070260 (HITBC) | China, Sichuan | FJ755412 | HQ697085 |  |
| Phoebe zhennan | Li L. 20070239 (HITBC) | China, Sichuan | HQ697212 | HQ697160 |  |
| Litsea auriculata | Li J. & Li L. 20070195 | China, Zhejiang (HITBC) | HQ697217 | HQ697174 |  |
| Litsea verticillata | Li L. & Wang Z. H. 20070337 (HITBC) | China, Hainan | HQ697218 | HQ697175 |  |
| Lindera erythrocarpa | Li J. & Li L. 20070203 (HITBC) |  | HQ697215 | HQ697167 |  |
