# Supplementary material for: Two regions, two lineages: Untangling two centuries of confusion between Machilus glaucescens and M. macranthus (Lauraceae) through integrative taxonomy
Source: PLoS One. 2026 Sep 9;21(9):e0357315. doi: 10.1371/journal.pone.0357315 (PMC13557376; doi:10.1371/journal.pone.0357315)
Supplement: Supplementary file 2 [file pone.0357315.s003.docx]

**Supplementary Information S3**

Table S3. The best-fit nucleotide substitution model of the two markers for both BI and ML analyses.

| Analysis | nr*ITS* | nr*LEAFY* |
| --- | --- | --- |
| ML | TPM2u+F+I+G4 | HKY+F+G4 |
| BI | HKY+I+G | HKY+G |
