# Supplementary material for: Two regions, two lineages: Untangling two centuries of confusion between Machilus glaucescens and M. macranthus (Lauraceae) through integrative taxonomy
Source: PLoS One. 2026 Sep 9;21(9):e0357315. doi: 10.1371/journal.pone.0357315 (PMC13557376; doi:10.1371/journal.pone.0357315)
Supplement: Supplementary file 3 [file pone.0357315.s006.docx]

**Supplementary Information**

**Fig S1.** Bar graphs depicting the percentage contribution of A) categorical and morphometric characters in the Dim-1 B) morphometric and categorical characters in the Dim-2 C) morphometric and categorical characters in the Dim-3 D) morphometric and categorical characters in the Dim-4. The dashed red color line represents the expected average contribution if all data types contributed equally.
