# Supplementary material for: Two regions, two lineages: Untangling two centuries of confusion between Machilus glaucescens and M. macranthus (Lauraceae) through integrative taxonomy
Source: PLoS One. 2026 Sep 9;21(9):e0357315. doi: 10.1371/journal.pone.0357315 (PMC13557376; doi:10.1371/journal.pone.0357315)
Supplement: Supplementary file 4 [file pone.0357315.s007.docx]

**Supplementary Information**

**Fig S2.** The complete Bayesian tree based on nr*ITS* and *LEAFY* sequences showing the phylogenetic positions of *M. glaucescens* and *M. macranthus* within the Persea group. Numbers at the nodes indicate Posterior probability (PP) values.
