# Supplementary material for: Two regions, two lineages: Untangling two centuries of confusion between Machilus glaucescens and M. macranthus (Lauraceae) through integrative taxonomy
Source: PLoS One. 2026 Sep 9;21(9):e0357315. doi: 10.1371/journal.pone.0357315 (PMC13557376; doi:10.1371/journal.pone.0357315)
Supplement: Supplementary file 6 [file pone.0357315.s009.docx]

**Supplementary Information**

**Fig S4.** (A) Predicted habitat suitability of *Machilus glaucescens* (B) Predicted habitat suitability of *Machilus macranthus.*

A

B
