# Supplementary material for: Two regions, two lineages: Untangling two centuries of confusion between Machilus glaucescens and M. macranthus (Lauraceae) through integrative taxonomy
Source: PLoS One. 2026 Sep 9;21(9):e0357315. doi: 10.1371/journal.pone.0357315 (PMC13557376; doi:10.1371/journal.pone.0357315)
Supplement: Supplementary file 7 [file pone.0357315.s010.docx]

**Supplementary Information**

**Fig S5.** Receiver Operating Characteristic (ROC) curve generated from one of the 10-fold cross-validation runs of the MaxEnt model for A) *M. glaucescens* and B) *M. macranthus*.

A

B
